# Assessing the Impacts of Correlated Variability with Dissociated Timescales

**DOI:** 10.1523/ENEURO.0395-18.2019

**Published:** 2019-03-20

**Authors:** Toshiyuki Takahashi, Yoshiko Maruyama, Hiroyuki Ito, Keiji Miura

**Affiliations:** 1 Department of Bioscience, School of Science and Technology, Kwansei Gakuin University, Hyogo 669-1337, Japan; 2 National Institute of Technology, Hakodate College, Hokkaido 042-8501, Japan; 3 Faculty of Information Science and Engineering, Kyoto Sangyo University, Kyoto 603-8555, Japan

**Keywords:** decoding analysis, information geometry, noise correlations, population codes, primary visual cortex, spontaneous activity

## Abstract

Despite the profound influence on coding capacity of sensory neurons, the measurements of noise correlations have been inconsistent. This is, possibly, because nonstationarity, i.e., drifting baselines, engendered the spurious long-term correlations even if no actual short-term correlation existed. Although attempts to separate them have been made previously, they were *ad hoc* for specific cases or computationally too demanding. Here we proposed an information-geometric method to unbiasedly estimate pure short-term noise correlations irrespective of the background brain activities without demanding computational resources. First, the benchmark simulations demonstrated that the proposed estimator is more accurate and computationally efficient than the conventional correlograms and the residual correlations with Kalman filters or moving averages of length three or more, while the best moving average of length two coincided with the propose method regarding correlation estimates. Next, we analyzed the cat V1 neural responses to demonstrate that the statistical test accompanying the proposed method combined with the existing nonstationarity test enabled us to dissociate short-term and long-term noise correlations. When we excluded the spurious noise correlations of purely long-term nature, only a small fraction of neuron pairs showed significant short-term correlations, possibly reconciling the previous inconsistent observations on existence of significant noise correlations. The decoding accuracy was slightly improved by the short-term correlations. Although the long-term correlations deteriorated the generalizability, the generalizability was recovered by the decoder with trend removal, suggesting that brains could overcome nonstationarity. Thus, the proposed method enables us to elucidate the impacts of short-term and long-term noise correlations in a dissociated manner.

## Significance Statement

The proposed measure for spike-count noise correlations, based on the local temporal detrending, enables us to decompose the correlated responses into long-timescale and short-timescale components. The proposed method is essential to elucidate the population codes in the era of large-scale electrophysiology as it works for large number of simultaneously recorded neurons while existing methods do not. With the additional help of the machine learning that classifies stimuli from neural activities, we demonstrate proper ways to assess the impacts on decoding of the presence of short-term or long-term noise correlations, separately. The well-designed decoding analysis with dissociated correlated activities will help to gain insight into the brain’s decoding strategies under changing environments.

## Introduction

The impacts and mechanisms of correlations in noises, i.e., trial-to-trial variations in neural responses to the same stimulus, have been issues in neuroscience (Cohen and Kohn, 2011; Doiron et al., 2016). The information theoretic studies showed that correlation in response noises can be a major determinant for coding capacities of sensory information by neurons (Abbott and Dayan, 1999; Sompolinsky et al., 2001; Miura et al., 2012; Latham and Roudi, 2013; Moreno-Bote et al., 2014). In some cases, even in a simple homogeneous network with tiny noise correlations, having more neurons does not help at all (Zohary et al., 1994; but see also Abbott and Dayan, 1999; Sompolinsky et al., 2001; Miura, 2012; Moreno-Bote et al., 2014). Therefore, it is extremely important to estimate noise correlations accurately in the era of large-scale electrophysiology (Steinmetz et al., 2018).

Although significant noise correlations have been observed in almost all recorded cortical areas, it has been pointed out that nonstationarity such as drifts in signals can engender artificial correlations even if no actual correlation exists (Bair et al., 2001; Ecker et al., 2010; Renart et al., 2010). Therefore, it is desired to dissociate the observed noise correlations into short-term and long-term components, where the latter is possibly caused by the background trends or fluctuations of the baseline activity (Fiser et al., 2004; Ikegaya et al., 2004; Sasaki et al., 2007; Luczak et al., 2015; Okun et al., 2015). Although attempts to separate them and estimate purely short-term noise correlations under changing environments have been made previously, they were *ad hoc* and applicable only to specific cases (Bair et al., 2001; Mitchell et al., 2009; Ecker et al., 2010; Renart et al., 2010). Even the latest Bayesian method requires considerable numbers of simultaneously recorded neurons as well as exponential computational costs to estimate instantaneous activities (Ecker et al., 2014; Rosenbaum et al., 2017). Thus, the estimation method, which requires only the recording of a pair of neurons and works for arbitrary baseline drifts nonparametricaly (Amari and Cardoso, 1997), is desired.

In addition to measuring the noise correlations, assessing their impacts is also very important. The degree to which sensory information is represented reliably by neural responses has been characterized by applying a decoding approach in a stochastic stimulus–response framework (Dayan and Abbott, 2001; Averbeck et al., 2006; Sharpee, 2017). That is, the decoding success rates can be used as a measure of accuracy of neural representations. One can take different features of neural activities as clues for decoding to see which feature carries information. Therefore, it is ideal, within this framework, if the dissociation of short-term and long-term correlations gives us a novel way to assess their respective impacts on information representations.

In this article, we propose an information-geometric method to unbiasedly estimate pure short-term noise correlations irrespective of the background brain activities. One effective way to use the information geometry, that generally finds orthogonal statistical parameters (Amari and Nagaoka, 2001; Miura, 2011; Amari, 2016), is to estimate only finite parameters of interest irrespectively of the other infinite numbers of parameters (Miura et al., 2006a,b, 2007; Miura and Uchida, 2008). Here, we used this infinite-dimensional scheme (Amari and Kawanabe, 1997; Miura, 2013) to dissociate the parameter for short-term correlation from the infinitely many parameters for (all possible) long-term baseline drifts nonparametrically. This allows us to estimate pure short-term correlations whatever the baseline drift is without demanding considerable numbers of simultaneously recorded neurons and high computational costs. Then, the accompanying statistical test as well as the existing nonstationarity test enabled us to dissociate short-term and long-term correlations. First, as benchmark simulations, we demonstrated that the proposed estimator is more accurate and computationally efficient than the conventional correlograms and the residual correlations with Kalman filters or moving averages of length three or more, while the best moving average of length two coincided with the propose method regarding correlation estimates. Next, when we excluded the spurious noise correlations of purely long-term nature, only a small fraction of V1 neuron pairs showed significant short-term noise correlations, possibly reconciling the previous inconsistent observations on the existence of significant noise correlations. Finally, with the additional help of the machine learning that classifies stimuli from neural activities, we assessed the impacts on decoding of the presence of short-term or long-term noise correlations, separately. The presence of pure short-term correlations slightly improved the decoding accuracy, while the pure long-term correlations deteriorated the generalization ability. However, the decrease in decoding accuracy by the long-term correlations was recoverable by using the decoder with offset, suggesting that the brain could overcome nonstationarity by detrending. Thus, our method enables us to elucidate the impacts of short-term and long-term correlations in a dissociated manner, advancing a modern, component-wise information theoretic analysis (Schneidman et al., 2003; Latham and Nirenberg, 2005; Averbeck et al., 2006; Sharpee et al., 2006).

## Materials and Methods

All the simulations and data analyses in this article were done by using R. Throughout the analyses in the article, the firing rate for each trial was used as an activity feature. The firing rate was computed as the spike count divided by the trial duration with a visual stimulus, which varied by trials from 1.0 to 1.7 s. Thus, when we say correlation coefficients or (trial-shifted) correlograms, we solely consider spike count noise correlations.

### Proposed estimator for short-term noise correlation

As a measure of short-term noise correlations, we proposed and used the following estimator,

(1) ρ^=Σ^12Σ^11Σ^22

where the covariances Σ^ij are estimated as

(2) Σ^11=2N∑t=1N/2{(x2t−1−x(2t)¯)2+(x2t−x(2t)¯)2}Σ^22=2N∑t=1N/2{(y2t−1−y(2t)¯)2+(y2t−y(2t)¯)2}Σ^12=2N∑t=1N/2{(x2t−1−x(2t)¯)(y2t−1−y(2t)¯)+(x2t−x(2t)¯)(y2t−y(2t)¯)}.

There in, *x_t_*and *y_t_*denote the neural responses in spike counts within a few seconds in the *t*-th trial, while the local mean activities were defined by

(3) $$\overset{¯}{x_{\left(2t\right)}}=\frac{x_{2t-1}+x_{2t}}{2}\;\;\;\;\text{and}\;\overset{¯}{y_{\left(2t\right)}}=\frac{y_{2t-1}+y_{2t}}{2}.$$

The proposed measure in Equation 1 is comparable to the conventional correlation coefficient. When we plotted in the form of correlograms, we first shifted one of two time series by *τ* trials and then computed the proposed measure for them.

### Code accessibility

The R code for computing the proposed correlation coefficient and its *p* value, as defined below in Statistical tests for short-term noise correlations, is freely available online at https://github.com/toshi-0415/eNeuro. The code is ready to run just by replacing the example data for Figure 4 with users’ own data.

As there can be a minor style difference in coding the proposed measure, we unified the rule and adopted the one with the minimum errors throughout the article and the downloadable code. That is, there are two possible ways for pairing two neighboring trials, (1) starting at the first trial as {3,4}, {5,6}, and (2) starting at the second trial as {2,3}, {4,5}, {6,7}, *…*. In the adopted {1,2} style, we took the average of the two estimated covariances, because we found it had smaller variances (estimation errors). This style difference only negligibly modifies the results and the overall conclusions never change.

### Assumption and derivation of proposed estimator

The proposed estimator in Equation 1 was derived for estimating parameters in a semiparametric statistical model. That is, the activities of two neurons were hypothesized to obey the following statistical model (Eq. 4) and the proposed estimator estimates the Gaussian covariance therein. Although the derivation and concise benchmark simulations were already shown elsewhere (Miura, 2013), the application to the real experimental data has not been done yet.

In this article, we solely consider the spike count within a trial, where the spiking activity of a neuron is integrated over a couple of seconds and, thus, well approximated by a Gaussian distribution. This leads us to consider a bivariate normal distribution for activities of two neurons, *q*(*x*, *y*; *μ_x_*, *μ_y_*, Σ), where *μ_x_*and *μ_y_*denote the means for two neurons’ activities and Σ denotes the covariance matrix. The activities *x* and *y* denote the spike counts of two neurons for a trial. These analyses address the situation in which the covariance matrix Σ is constant whereas the signals *μ* can change over time. Especially, when the signals *μ* are distributed randomly, but two consecutive signals are the same from continuity condition, the distribution of activities at time 2*t−*1 and 2*t* (*t* = 1, 2,*…*) can be described as a mixed model,

(4) $$p(\{x_{2t-1},y_{2t-1},x_{2t},y_{2t}\};\Sigma ,k(\mu_{x},\mu_{y}))=\int k(\mu_{x},\mu_{y})\times q(x_{2t-1},y_{2t-1};\mu_{x},\mu_{y},\Sigma )q(x_{2t},y_{2t};\mu_{x},\mu_{y},\Sigma )d\mu_{x}d\mu_{y}$$

where *k*(*μ_x_,μ_y_*) denotes an unknown distribution of the signals. The only assumption made here is that the consecutive signals have equal value, at least approximately (see the practical discussion below, at the end of Optimality of proposed estimator from statistical viewpoint). That assumption is minimal and realistic as it is satisfied, e.g., when the signal drift is continuous, and preferably, sufficiently low. From another viewpoint, this definition of noises as the activities which is not locally flat over time is quite convenient for estimation.

Furthermore, Equation 4 is a semiparametric model (Bickel et al., 1993; van der Vaart, 1998) because it has both a vector Σ and a function *k*(*μ_x_,μ_y_*) as parameters. It is generally not easy to estimate parameters in semiparametric models because a function space is fundamentally infinite dimensional (Neyman and Scott, 1948). However, it is known that, for some cases, only parameters of interest can be estimated efficiently through differential geometric methods on the manifolds of a family of probability distributions (Amari and Kawanabe, 1997; Amari and Nagaoka, 2001; Miura et al., 2006a,b, 2007; Miura and Uchida, 2008). For this model, it is possible to estimate the three constant parameters Σ = {Σ_11_,Σ_12_(= Σ_21_),Σ_22_} whatever the signal drift *k*(*μ_x_,μ_y_*) is.

After a lengthy calculation in Miura (2013), the estimator was obtained as in Equation 2. As the proposed estimator looks so simple, one might think that one can easily construct an arbitrary local smoother similar to the proposed estimator. However, because any arbitrarily invented estimators have larger estimation errors (biases and variances) in general, it is actually very difficult to discover an optimal estimator from scratch. As far as we know, other than information geometry, there is no systematic way to analytically derive an optimal estimator that works under arbitrary trends nonparametrically. Fortunately, it is very easy to just prove the optimality of the derived estimator, once it was derived. Therefore, we take advantage of this fact for the educational purpose in what follows. That is, we do not repeat the derivation but rather only check the answer and demonstrate the performance of the proposed estimator concisely in the following section.

### Optimality of proposed estimator from statistical viewpoint

Here, we summarize and prove the optimality property from the statistical viewpoint. Specifically, we show that the proposed estimator has no bias (i.e., correct on average) and minimum variances (i.e., smallest errors) among the estimators which work unbiasedly for arbitrary baseline drifts.

The unbiased nature of the proposed estimators is clear from the fact that the estimators in Equation 2 are normalized by dividing not by 2(= *M*) but by 1(= *M−*1). Normalization of this type is widely known to guarantee the unbiased estimation for the covariances of Gaussian distributions. In fact, with *X_t_*= (*x_t_,y_t_*) and *μ* = (*μ_x_,μ_y_*), the expectation of Σ^12 can be calculated as an integral over the probability distribution in Equation 4 as

(5) E[Σ^12]:=∫(∫Σ^12(X2t−1,X2t)q(X2t−1|Σ,μ)q(X2t|Σ,μ)×dX2t−1dX2t)k(μ)dμ=∫Σ12k(μ)dμ=Σ12.

This means that the estimator is unbiased or the estimator works (at least) “on average.” The variance of the estimate can be similarly computed as

(6) Var[Σ^12]:=∫(∫(Σ^12(X2t−1,X2t)−Σ)2×q(X2t−1|Σ,μ)q(X2t|Σ,μ)dX2t−1dX2t)k(μ)dμ=Σ11Σ22+Σ12Σ21.

Surprisingly, Σ^ has the minimum variance (= estimation error) among all the estimators. To prove this, assume that $\hat{\theta }(X_{2t-1},X_{2t})$ is an arbitrary estimator of Σ_12_, that is,

E(θ^(X2t−1,X2t)):=∫θ^(X2t−1,X2t)p(X2t−1,X2t;Σ,k(μx,μy))dX2t−1dX2t=Σ12.

Note that we assumed that the expectation is equal to the statistical parameter of interest because any estimator should work at least “on average.” By using the Cauchy–Schwartz inequality in functional space (|f||g|≥f·g) with f=θ^(X)−Σ12 and $g=\frac{d\log p(X)}{d\Sigma_{12}}$, we get

(7) Var[f]Var[g]=Var[θ^](Σ11Σ22+Σ12Σ21)−1

(8) ≥∫fg p(X)dX=∫d((θ^(X)−Σ12)p(X))dΣ12dX−∫d(θ^(X)−Σ12)dΣ12p(X)dX=1,

where *X* = (*X*_2_*_t_*_-1_, *X*_2_*_t_*). This shows that any estimator θ^ of Σ_12_ at least have the minimum variance in the right-hand side:

(9) Var[θ^]≥Σ11Σ22+Σ12Σ21.

Actually, the proposed estimator Σ^12 attains this minimum variance as shown in Equation 6. Thus, for any estimator θ^,

(10) Var[θ^]≥Var[Σ^12].

Similar relations hold for Σ_11_ and Σ_22_.

We have demonstrated that the proposed estimator is optimal as far as the assumption on the statistical model hold. Practically, due to the violation of the assumption that the consecutive two signals (means) are exactly the same, the biases can arise. However, it can be shown from a simple calculus that the biases are generally small. In fact, if the consecutive signals are

(11) $$\text{E}[x_{2t-1}]=\mu -\epsilon \;\;\;\text{and}\text{E}[x_{2t}]=\mu +\epsilon ,$$

differing of order of *ϵ*, then, the biases are of the second order of *ϵ*:

(12) E[Σ^11]=E[(x2t−1−x2t)22]=σ2+2ϵ2.

Thus, even if one assumes that the biases accumulate over the time points whose size is of order $\frac{1}{\epsilon }$, the total bias is still negligible, being of order $\epsilon^{2}\times \frac{1}{\epsilon }=\epsilon$. This suggests that even if the signal drifts slowly O(ϵ) as in Equation 11, keeping the difference between the first and the last activities finite *O*(1) after a long time sequence $O(\frac{1}{\epsilon })$, the total bias is negligibly small *O*(*ϵ*). In fact, Figure 5*D* demonstrates that the proposed statistical test detects no spurious short-term correlations even if signals drift in the real V1 data.

### Simulation of activities of two neurons with drifting baselines

**Figure 1.**
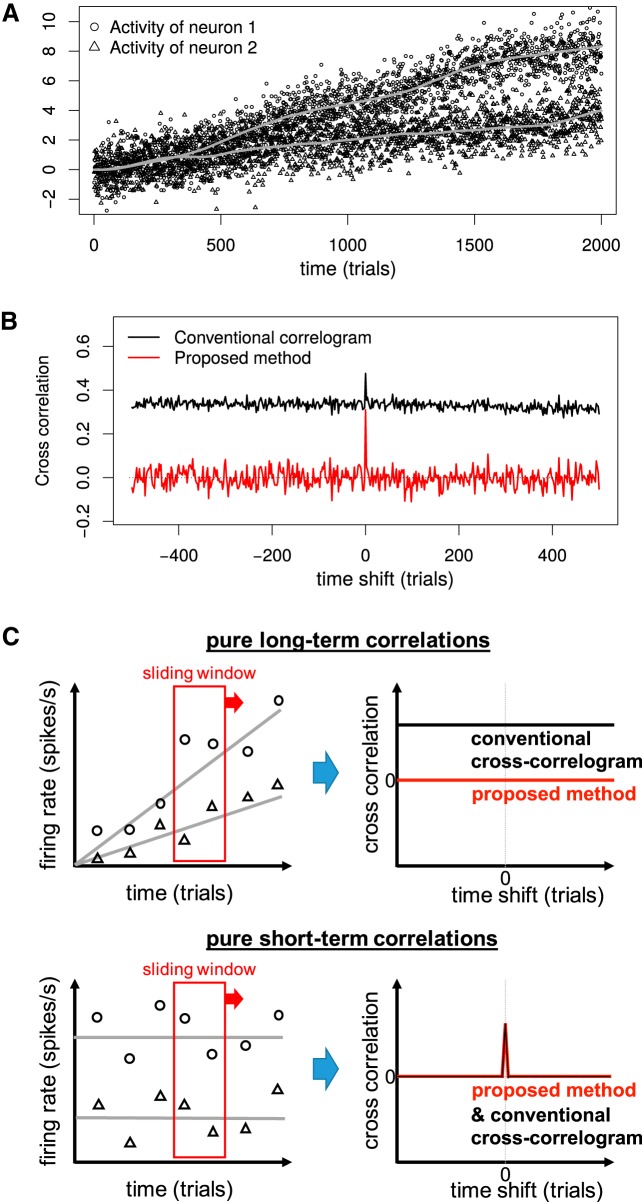
Comparison of conventional cross-correlogram and proposed method. ***A***, Artificial activities of two neurons, simulated as sums of baselines and trial-to-trial noises. The thick gray smooth curves denote time-dependent baselines μ generated by the ARIMA(0,2,1) model, on which the bivariate Gaussian noises were added to generate the neural activities. The added noises have significant spatial or interneuronal correlations but no temporal correlation because intertrial intervals are assumed to be fairly long (≥3 s). ***B***, The estimated cross-correlations for the simulated activities in ***A*** by the proposed method (red) and the conventional correlogram (black). Only the proposed method works and shows a proper peak at the origin. ***C***, Schematic illustrations of how the proposed method works for the cases with pure long-term or short-term correlations. The cross-correlation computed within each local window, where the baselines are instantaneously constant, are averaged across sliding windows to capture only short-term correlations whatever the baseline drift is.

The simulations of bivariate Gaussian noises added to the baselines generated by the ARIMA models for activities of two neurons in Figure 1*A* were performed with **mvrnorm()** and **arima.sim()** functions in R.

### Conventional cross-correlograms

The conventional cross-correlograms were computed with **cor()** function in R for the manually trialshifted data. As the function returns NA (i.e., not available) when either of two neurons show no spike across all 40 trials, we excluded those pairs from the analyses in the article. Note that the proposed method also returns NA for those pairs. We computed time-shifted noise correlations or cross-correlation functions separately for different stimuli, because recent works indicated the stimulus dependency of noise correlations (Kohn and Smith, 2005; Maruyama and Ito, 2013; Ruff and Cohen, 2016).

### Kalman filter method

The smoothing by the Kalman filter to obtain the baseline trend of the simulated neural activities was computed with **dlmFilter()** function in **dlm** package for R (Petris et al., 2009). The noise correlations in residuals was obtained by the maximum likelihood method for data fitting with **dlmMLE()** function in the same package. The statistical model for the baseline trend *μ_t_*we assumed to decode with Kalman filter was

(13) $$\mu_{t+1}=F\mu_{t}+\eta (\text{Gaussian noise})X_{t}^{\left(i\right)}=G^{\left(i\right)}\mu_{t}+\xi_{i}(\text{Gaussian noise})$$

where *X* ^(^*^i^* ^)^ denotes *i*-th neuron’s activity and F and G are to be estimated by data fitting.

The computational time was measured by **proc.time()** function in R on iMac with 3.3 GHz Intel Core i5 and 32-GB memory.

### Statistical tests for short-term noise correlations

We detected neuron pairs that have significant short-term noise correlations by using the statistical test accompanying our estimator. As is usual with statistical tests, we computed *p* values under the null hypothesis of no correlation.

One possible way, which we did not adopt, was to assume the asymptotic normality for the distribution of the proposed estimator, whose mean and variance can be computed from Equations 5, 6 (or from simulations). However, for the current case, each neuron has only finite 40 trials per stimulus, and thus, the normality assumption holds only approximately. Therefore, for example, the control *p* value distribution for the one-time-shifted data are not as flat as in Figure 5*D*, although it is approximately flat. Although this method saves the computational time, it seems to lack the accuracy in *p* values.

To pursue the full accuracy, we resorted to the computational method with the white Gaussian Monte Carlo simulations for reference activities of neuron pairs. Here, the test was based on the idea that even if there is no short-term correlation, its estimate from finite 40 trials takes a non-zero value (error), which varies according to some statistical distribution. First, we obtained the shape of the distribution as accurate as possible by repeating the Monte Carlo simulations a million times. Next, the *p* value for a given estimate is defined as its percentile in this numerically obtained distribution. That is, the *p* value is defined so that the *p* value distribution is completely flat for white Gaussian noises. To be precise, the *p* value varies by realization of the activities of two neurons, but, with many realizations, one obtains the uniform distribution for the *p* values. Note that the uniform *p* value distribution is a hallmark of a good statistical test. Finally, if an estimate is too high or too low within the numerically-obtained distribution (typically top 2.5% for both sides, corresponding to positive and negative correlations), it is detected as significant or violating the null hypothesis.

**Figure 2.**
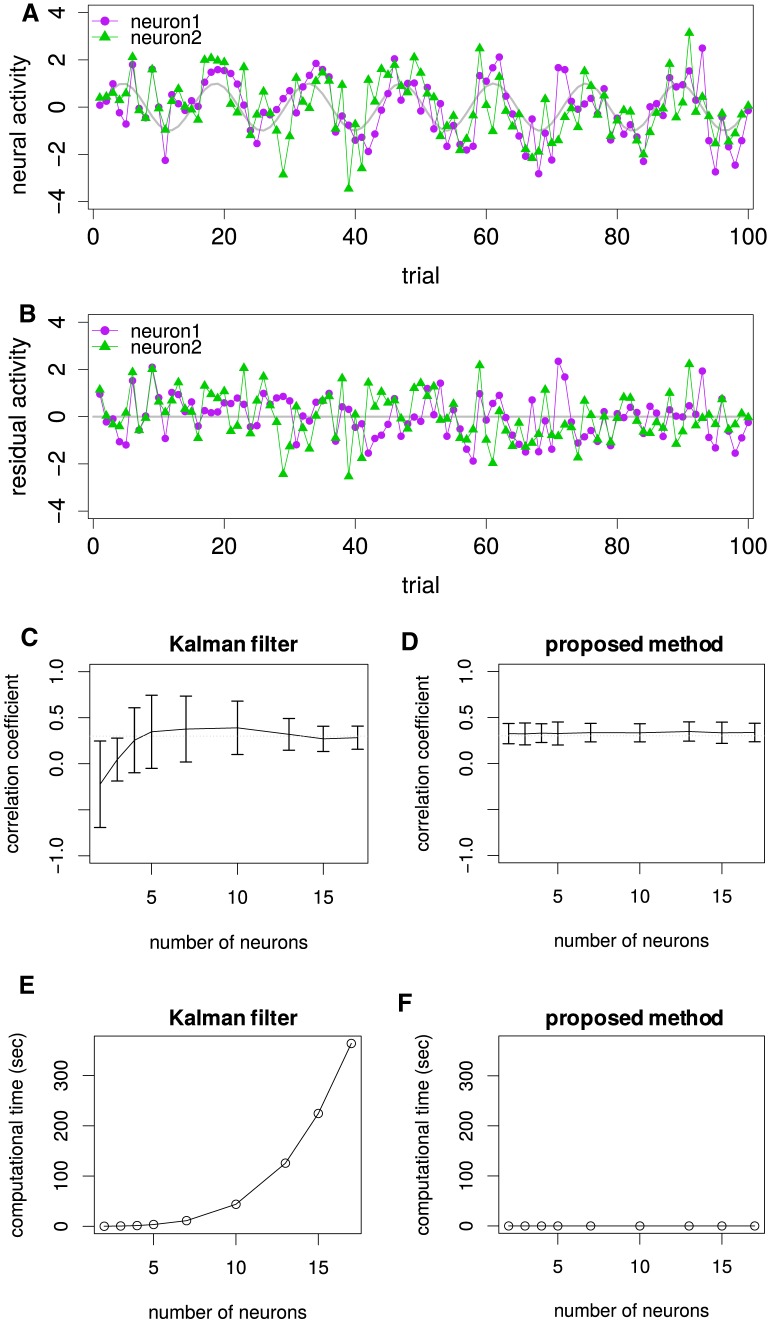
Comparison of conventional Kalman filter method and proposed method. ***A***, The simulated activities of two neurons (red and blue) for 100 trials with the common sinusoidal baseline trend. The thick gray line denotes the model trend used for the data generation. The activities of two neurons at each time are generated as the sum of the baseline trend and the bivariate Gaussian noises with unit variances and 0.3 correlation coefficient. When we simulated more than two neurons simultaneously, the additional neurons shared the trend but did not have noise correlation (data not shown). Thus, among *N* simulated neurons, only neurons 1 and 2 have non-zero correlation coefficient, which is to be estimated. ***B***, The residual activities after the removal of the estimated trend by the Kalman filter from the activities in ***A***. ***C***, The noise correlations in the residuals averaged across 100 realizations of the simulated data. The horizontal dotted gray line for the true correlation coefficient (=0.3) indicates that the conventional Kalman filter method does not work when the number of simultaneously simulated neurons are small. The error bars representing the SD demonstrate the large trial-to-trial variability in the results. ***D***, Noise correlations estimated by the proposed method from the same data. The horizontal dotted gray line for the true correlation coefficient (=0.3) indicates that the proposed method always works. The error bars representing the SD demonstrate the small variability in the results. ***E***, The computational time for the conventional Kalman filter method. ***F***, The computational time for the proposed method.

When we computed the control *p* value distribution for “one-time-shifted” data in Figure 5*D*, we actually shifted two-trials. This is because our proposed estimator treats the time series by pairs of time points as in Equations 2, 4. This is also why we shifted 2, 4, 6, …, trials in Figures 1, 2.

The R codes for computing the proposed short-term correlation and the accompanying statistical test was handwritten.

As the level of significance,$\frac{0.01}{\left(16\frac{N(N-1)}{2}\right)}$, where *N* denotes the number of neurons in the session and 16 is for 16 stimuli, was entirely used in the article (specifically in Fig. 6). That is, we employed Bonferroni’s multiple comparison technique, because we wanted to keep the number of neuron pairs moderately. Note that if we remove a neuron, we lose many pairs in the same session.

### Statistical tests for nonstationarity

We selected neurons with and without nonstationarity by using the serial correlation test for randomness of fluctuations (CASE64 in Kanji, 2006). To remove the effect of stimulus presentation from the time series of neural activities, we averaged local 16 trials within a single block where 16 different stimuli are presented pseudo-randomly. In this way, the length of the time series was reduced from original 640 to 40 trials, to which we applied the test. The R code for the test was handwritten. The validity of the test was confirmed by the observation that the test returns uniformly distributed *p* values for the Gaussian white noises or the completely random time series in which a random number is generated according to the normal distribution at every time. Note that the resulting *p* value varies by (random) time series and, here, we confirmed that the distribution got flat with many realizations.

As the level of significance, 0.01 was entirely used in the article (specifically in Figs. 5, 6). We did not employ the multiple comparison techniques, as we wanted to categorize suspicious neurons into the nonstationary neuron pool, conservatively.

### Classification analysis and principal component analysis

For the classifications of 16 visual stimuli based on the firing rates of neurons, we solely used **lda()** function in R in this article, although the result did not change significantly when we used the support vector machine. The classification was done session by session to use the simultaneity of the recorded data. For the statistical significance, the means of classification success rates for all sessions were compared between different conditions by the paired *t* test. Only the sessions with more than five neurons remaining after the selections by short-term or long-term correlations were included in the classification analyses for reliability.

For the principal component analysis, we used **prcomp()** command in R. As a preprocessing, we first averaged the neural responses to each stimulus, in order not to include the trial-to-trial variability in the visualization by principal components. That is, we essentially visualized the tuning curves. In addition, here we did not standardize the activity of each neuron or tuning curve, because we did not want to enlarge small noises within bad neurons who do not respond to any stimuli at all. That is, not to listen to purely noisy neurons too much, we did not enlarge the tuning curves even if their amplitudes are small. In Figure 6*B*, the same neuron pool as in Figure 6*C*, right, i.e., the neurons with pure long-term correlations, was used (189 neurons from 23 sessions).

### V1 neuronal spikes

The experimental details for the cat V1 anesthetized recordings we reanalyzed have been previously described (Maruyama and Ito, 2013, 2017). Briefly, 566 neurons were recorded in 48 sessions with 640 trials (40 repeats of 16 visual stimuli) from five adult male cats. Two types of electrode arrays were adopted for the recordings: a four-tetrode array and an array of eight single microelectrodes, both of which were fabricated in the laboratory.

The eyes were focused on the tangential screen at a distance of 57 cm using the tapetal reflection technique and an appropriate set of gas-permeable contact lenses. The pupils were dilated using phyenylephrine hydrochloride (Neosynesin eye solution). All animal procedures were performed in accordance with the Kyoto Sangyo University animal care committee’s regulations.

Once stable recordings were obtained, the receptive field properties (location) of the multi-unit activity recorded by each electrode were mapped, using a mouse-controlled moving light bar presented on a 21-inch color monitor (1024 × 768 resolution, vertical refresh rate of 80 Hz) at a distance of 57 cm from the eyes. Because the receptive fields of the units recorded by the high-density electrode arrays had significant overlap, the units were stimulated by moving the light bars on a dark background crossing over the region covering all of the receptive fields. The stimuli consist of the light bars of 16 orientations equally spaced (i.e., with an angular separation of 22.5°) that move along the direction of the normal. We ran 40 trial blocks in which each of the 16 stimuli were presented in a pseudo-random order with an intertrial interval of 3 s. The bars traveled an angular distance of 3–5° over a period of 1.0–1.7 s (speed 3°/s).

Multi-unit activities recorded by each electrode were sorted to recover the activities of individual single units using custom spike sorting software (Gray et al., 1995).

## Results

For the purpose of measuring the spike-count noise correlations in different timescales and assessing their respective impacts on neural representations, we used the novel information geometric estimators of pure short-term correlations, which can be dissociated from long-term correlations in a nonparametric manner, that is, whatever the baseline drifts are. Before we applied this proposed method to the neural responses in V1, we checked whether and how it worked for the simulated time series as a benchmark.

### Proposed estimator works irrespective of baseline drifts

First, we randomly generated the artificial time series which mimics the activities of two neurons, whose baselines drift across many trials. Note that nonstationarity, often observed experimentally in an unreproducible manner, was indispensable for the simulation, as we wanted to see whether the proposed method can overcome it. In the numerical simulation in Figure 1*A*, the activities of two neurons were created by adding the bivariate Gaussian noises to the smoothly drifting trends, which, in turn, were independently generated for the two neurons by ARIMA(0,2,1) model whose moving average coefficient was 0.6 (Harvey, 1993). Here, a significant short-term noise correlation (ρ = 0.3) was induced only between simultaneous noises for two neurons, mimicking typical neuroscience experiments where significant trial intervals of seconds order wash out intertrial temporal correlations in spike counts. An example realization of the simulation in Figure 1*A*, that mimics one recording session, shows hallmark drifting baselines, which is definitely unreproducible and hard to estimate with limited sample number or from this “single snapshot” data. Note that here we exclusively consider trials as a unit for time axes, instead of fine scale windows such as 1-ms bins.

Figure 1*B* shows the cross-correlation functions for the realization of simulated activities for two neurons in Figure 1*A* computed by both the conventional correlogram and the proposed method (Eqs. 1, 2). Here, the correlation coefficient ρ was estimated for each time-shifted data, where the activities of one neuron was time-shifted while those for the other neuron was kept. Because of the wrong assumption of the constant baselines, the conventional correlation coefficients caused a broad cross-correlation function attributable to the temporal correlations in the baselines. That is, the correlation coefficient is positive because when the activity of neuron 1 is higher (lower) than its average at a late (early) trial, that of neuron 2 is also higher (lower). Adding a time shift does not affect this situation as there is a global trend in Figure 1*A*. Note that broad cross-correlation functions have been observed for the experimental data (Bair et al., 2001). On the other hands, the proposed method gave a satisfiable result, correctly causing 0 for the time shifted data and the short-term correlation ρ (=0.3) for the simultaneous data as demonstrated by a clear peak in Figure 1*B*. Note that the estimated correlation coefficient $\hat{\rho }(\approx 0.3)$ is not only useful for statistical tests but also interpretable as a simultaneous covariation of Gaussian noises because our method is statistical model-based.

The reason for the flexible estimation by the proposed method is that it estimates the covariance for two neurons within each local window, where the background activity is assumed to be almost constant, and, then, averages the local estimates across sliding windows as in Figure 1*C*. Note that our method is based on the assumption that the short-term correlation (or the covariance parameter of Gaussian noises) is constant over time. Consequently, the proposed method enables estimation of the short-term correlations existing in the simultaneous activities independently of the drifting baselines. Figure 1*C* shows how this method works for the cases with pure long-term (Fig. 1*C*, top) or short-term (Fig. 1*C*, bottom) correlations. In the case of pure long-term correlations in Figure 1*C*, top, the estimate of the correlation in the short window is zero (on average), as there is no real short-term correlation and the baseline drift is negligible in this short timescale. Note that an implicit assumption in the proposed method is that within a short window, the baseline drift is absent or negligible, although the violation of this assumption, if small enough, actually does not matter (Materials and Methods). In the case of pure short-term correlations in Figure 1*C*, bottom, the estimate of the correlation in the short window is non-zero (on average), as there is a real short-term correlation although the baseline drift is absent. In this way, the proposed “local” estimates, that can be unaffected by the slow, long-term trends, work fairly well even if the baseline activities drift arbitrarily over time.

### Proposed estimator requires less neurons and computational powers than conventional Kalman filters

The key idea for the proposed estimator of noise correlations resides in the local detrending. However, there are other types of detrending methods such as Kalman filters. The latest studies also computed the correlations in residuals after the neural activities were smoothed and detrended by the Kalman filter-like methods (Ecker et al., 2014; Rosenbaum et al., 2017). Therefore, we performed another benchmark simulation to compare the conventional Kalman filter method with the proposed method. Specifically, we checked whether the two methods work in the presence of sinusoidal baseline drifts in simulations.

Figure 2*A* shows the activities of two neurons, simulated as the time series of length 100 with the common sinusoidal baseline trend. The activities of two neurons at each time are generated as the sum of the baseline trend and the bivariate Gaussian noises with unit variances and 0.3 correlation coefficient. When we simulated more than two neurons simultaneously, the additional neurons shared the trend but did not have noise correlations. Thus, among *N* simulated neurons, only neurons 1 and 2 have a non-zero correlation coefficient, which is to be estimated.

Figure 2*B* shows the residual activities after the removal of the estimated trend by the Kalman filter from the activities in Figure 2*A*. The dark horizontal line indicates the estimated trend, which has been already removed from the activities.

Figure 2*C* shows the noise correlations in the residuals averaged across 100 realizations of the simulated data. The horizontal dotted gray line for the true correlation coefficient (=0.3) indicates that the conventional Kalman filter method does not work when the number of simultaneously simulated neurons are small. Naturally, recording from more neurons helps to estimate the current baseline trend, which is essentially the average activities of neurons in this easiest situation. If one does not know baseline trends accurately, the estimation of noise correlations fails as well. In more realistic situations, in which neurons do not necessarily share baseline trends, more neurons would be required to estimate the noise correlation by the Kalman filter-like methods.

Figure 2*D* shows the noise correlations estimated by the proposed method from the same data. The horizontal dotted gray line for the true correlation coefficient (=0.3) indicates that the proposed method always works. Note the proposed methods only requires the activities of the two relevant neurons as evident in Equations 1, 2.

Furthermore, the Figure 2*E* shows that the Kalman filter can be fairly expensive in computational time with as small as 15 neurons. Given the number of simultaneously recorded neurons is increasing rapidly, the computational costs can easily constitute a limiting factor. Thus, the proposed method is advantageous not only in the estimation accuracy, but also in the computational cost as demonstrated in Figure 2*F*.

The results obtained here are fairly general. Although the sinusoidal trend with seven cycles was entirely used in this article, qualitatively the same results were obtained for a wide range of numbers of cycles (4–10; data not shown). Imagine that the sinusoidal waves with different periods can exhaust the different possible timescales. In fact, it has been numerically demonstrated that the proposed method worked also for linear as well as stepwise trends in the previous work (Miura, 2013), although all these numerical simulations just confirmed the mathematical statement that the proposed method is robust against arbitrary drifts. Although the proposed method might look too easy at first glance, any other *ad hoc* estimators of covariances cannot achieve the unbiasedness (i.e., correctness) under arbitrary drifts. Moreover, although the latest best Bayesian methods can be regarded as variants of Kalman filter methods and some of them might improve the estimation accuracy slightly, we believe that the problem in computational costs is unavoidable in any case.

### Proposed estimator has less errors than conventional moving averages

**Figure 3.**
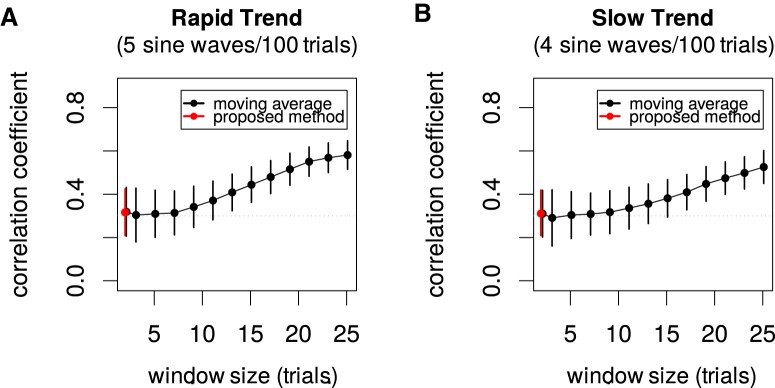
Comparison of conventional moving average method and proposed method. As in Figure 2, the simulated neural activities had the sinusoidal trend with five waves (***A***) or four waves in 100 trials (***B***). For the moving average method, the neural activities were first smoothed by the moving average with various window sizes and then the correlation coefficients were computed for the residuals. The mean ± SD of the estimated noise correlations across 100 realizations of the simulated data plotted. The horizontal dotted gray lines for the true correlation coefficient (=0.3) indicate that the biases are prominent for longer window sizes and for rapidly changing trends.

As some of the previous works (Cohen and Newsome, 2008; Mitchell et al., 2009) simply used the moving average for detrending, we next compared the conventional moving average method with the proposed method (Fig. 3).

In the comparison, as in Figure 2, the simulated neural activities had the sinusoidal trend with five waves (Fig. 3*A*) or four waves in 100 trials (Fig. 3*B*). For the moving average method, the neural activities were first smoothed by the moving average with various window sizes, and then the correlation coefficients were computed for the residuals. The horizontal dotted gray lines for the true correlation coefficient (=0.3) indicate that the biases are prominent for longer window sizes and for rapidly changing trends.

Although the moving average method is uniquely defined for odd window sizes, some variants can be considered when the window size is two (and even lengths in general). When the window size is two, however, one can carefully define the moving average method so that it coincides with the proposed method regarding the correlation coefficients. To be precise, the moving average method actually fails and underestimates both the variances (Σ_11_, Σ_22_) and the covariance (Σ_12_) by half, although the correlation coefficient as their ratio is intact as $\rho =\Sigma_{12}/\sqrt{\Sigma_{11}\Sigma_{22}}$. For example, when the true variances for the activities of two neurons are both 1 and the true covariance is 0.2, the moving average method on average estimates them as 0.5, 0.5, and 0.1 while the correlation coefficient estimated as their ratio coincides with that of the proposed estimator, which is always near 0.2.

Some previous works used longer window lengths for detrending (previous and future 20 trials for Cohen and Newsome, 2008; and Gaussian kernels with σ = 5 trials for Mitchell et al., 2009). Although it is not clear whether the actual drift is as drastic as in Figure 3, our message in this article is that, in fact, one can safely shorten the window length to the minimum size, i.e., two.

### Examples of noise correlations in V1 neuron pairs

**Figure 4.**
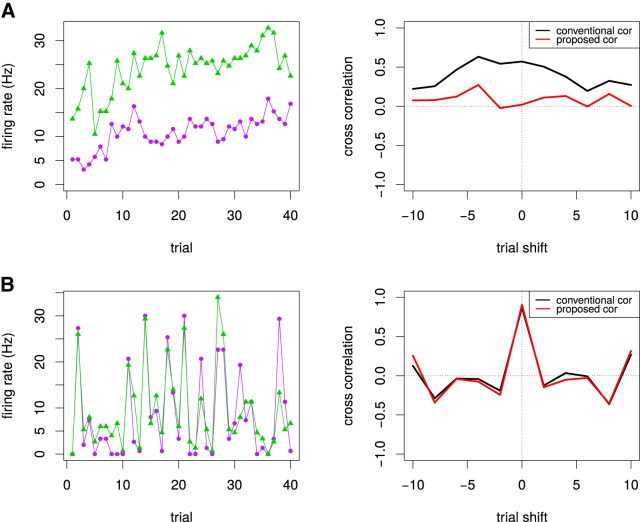
Examples of noise correlations for two V1 neuron pairs. ***A***, For the nonstationary case where the time series for the both neurons show significant drifts (left), the broad cross-correlation was estimated by the conventional cross-correlogram (*p* = 0.00012 at the origin) but no short-term correlation by the proposed method (*p* = 0.92 at the origin). ***B***, For the stationary case where the time series for the both neurons do not show significant drifts but the simultaneous activities tend to synchronize, the narrow cross-correlation at the origin was estimated by both the conventional correlogram (*p <* 10^5^) and the proposed method (*p <* 10^5^).

Here, we applied the proposed method for estimating pure short-term noise correlations to the pairs of the neural activities in the primary visual cortex. Figure 4 shows the interneuronal noise correlations of two example pairs of neurons estimated by the proposed method as well as the conventional cross-correlogram. We computed time-shifted noise correlations or cross-correlation functions. Note that we solely computed noise correlations for a fixed stimulus in this article, because recent works indicated the stimulus dependency of noise correlations (Kohn and Smith, 2005; Maruyama and Ito, 2013; Ruff and Cohen, 2016). For the putatively nonstationary neuron pairs in Figure 4*A*, the time series for the activities of both neurons showed significant drifts. The conventional correlogram showed the spurious correlations across wide shifts of trials, while the proposed method indicated no short-term correlation successfully. Note that similar broad cross-correlation functions have been observed previously (Bair et al., 2001). For the putatively stationary neuron pairs in Figure 4*B*, the time series for the both neurons did not show significant drifts but the simultaneous activities tended to synchronize. Both the conventional correlogram and the proposed method correctly detected the short-term noise correlation at the origin. Thus, the proposed method succeeded to clarify the fine structure of noises in real V1 data by detecting purely short-term correlations.

### Both long-term and short-term correlations are widely observed in V1

**Figure 5.**
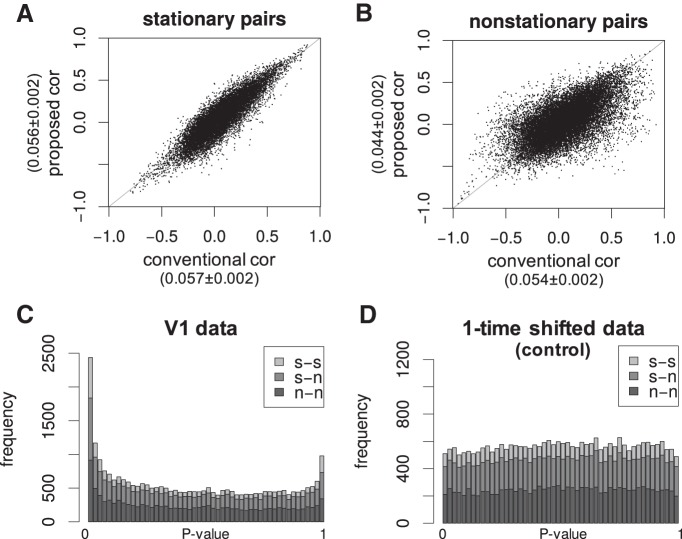
Population summary of noise correlations for all recorded V1 neurons. ***A***, The proposed short-term noise correlations plotted against the conventional correlation coefficients for all the simultaneously recorded pairs of stationary V1 neurons (*n* = 12,931 pairs). The stationary or nonstationary neurons were selected by the statistical serial correlation test for nonstationarity. The numbers along the axes denote the mean ± SEM. ***B***, Same plot for all the simultaneously recorded pairs of nonstationary V1 neurons (*n* = 18891 pairs). Note that the correlations are highly reproducible located along the diagonal for the stationary neuron pool but not reproducible for the nonstationary neuron pool, suggesting that the proposed method successfully removes long-term noise correlations by detrending. ***C***, The distribution of the *p* values for the statistical significance of the proposed short-term noise correlations for V1 data. *s-s* denotes the pair of two stationary neurons. *s-n* denotes the pair of stationary and nonstationary neurons. *n-n* denotes the pair of two nonstationary neurons. The non-uniformity of the distribution indicates that the significant short-term correlations for some pairs are not obtained by chance. ***D***, The control distribution of the *p* values for the same test obtained for the one-time-shifted V1 data that cannot have short-term correlations. The uniform distribution demonstrates that, desirably, the statistical test detects no spurious short-term correlation even if the signals drift in the V1 data. Note that, in contrast, the conventional correlogram in Figure 1*B* resulted in the non-zero correlations even for time-shifted data.

Next, we investigate the noise correlations for the entire population of pairs of simultaneously recorded neurons. Figure 5*A*,*B* plots the short-term noise correlations estimated by the proposed method against the conventional correlation coefficient for all the pairs within the stationary or nonstationary neurons. The stationary or nonstationary neurons were selected by the statistical serial correlation test for nonstationarity. In Figure 5*A*, for the stationary neuron pool, the correlations are highly reproducible, located along the diagonal line. Meanwhile, in Figure 5*B*, for the nonstationary neuron pool, they are not reproducible, scattered apart from the diagonal line, with smaller absolute values for the proposed method. The result suggests that the proposed method successfully removes long-term components of noise correlations essentially by detrending. Note that some of the smallest noise correlations reported in the previous works were obtained for the detrended time series (Bair et al., 2001; Ecker et al., 2010; Renart et al., 2010), consistent with our observation. Thus, the nonstationarity or a baseline drift may engender spurious correlations even if no actual short-term correlation exists.

Figure 5*C* shows the *p* value histogram for the statistical significance of the proposed short-term noise correlations for V1 data. The non-uniformity of the distribution indicates that the significant short-term correlations for some pairs are not obtained by chance. Furthermore, all types of pairs, irrespective of stationary and nonstationary neurons, show significant short-term correlations. As a control to check the validity of our statistical test, Figure 5*D* shows the *p* value histogram for the same test obtained for the one-time-shifted V1 data that cannot have short-term correlations. The resulting uniform distribution demonstrates that, desirably, the statistical test detects no spurious short-term correlation even if the signals drift in the V1 data. Remember, in contrast, the conventional correlogram in Figure 1*B* resulted in the non-zero correlations even for time-shifted data.

In total, significant fractions of noise correlations seem to be explainable by the long-term components while there are some pairs with significant short-term correlations as well. We next pursue whether each component is either helpful or harmful for the sensory information representation in the brain.

### Impacts of short-term and long-term noise correlations are dissociable

Finally, we assessed the impacts on decoding of the presence of short-term or long-term correlations, separately. Our estimator enables us to elucidate the impacts of short-term and long-term correlations in a dissociated manner, as we will see. Here, we performed the linear discriminant analysis of stimuli based on the neural responses and used the classification success rates as a measure of the accuracy of neural coding. That is, the higher the classification success rate is, the more accurate the neural coding should be.

**Figure 6.**
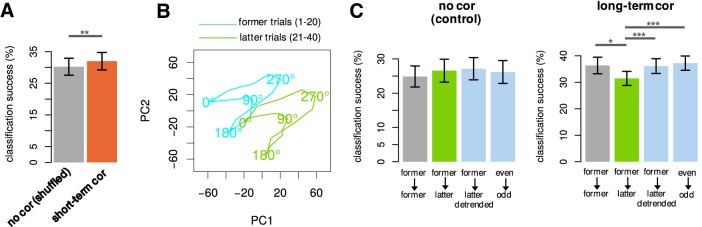
Impacts of short-term and long-term components of correlated activities of V1 neurons. ***A***, The classification success rates in the absence and presence of pure short-term correlations (mean ± SEM). In the presence of pure short-term correlations, the decoding accuracy was slightly improved (*p* = 0.0044, paired *t* test, 15 sessions with 134 neurons). Note that the chance level is $\frac{1}{16}(=6.25\%)$, as 16 stimuli were decoded. ***B***, The baseline drifts, which cause long-term correlations, were visualized by the principal component analysis for the average responses to 16 visual stimuli (tuning curves) of the neurons with significant baseline drifts (to be precise, the same neuron pool as in ***C***, right). The average responses for 1st–20th trials (turquoise blue) and 21th–40th trials (green) demonstrate that the entire activities of neurons shift over trials. ***C***, Decoding accuracy in the absence and presence of pure long-term correlations. The cross-validated classification success rates for four types of learning were compared: (1) when trained by former trials and tested by former trials, (2) when trained by former trials and tested by latter trials, (3) when trained by former trials and tested by latter trials after the respective global means were subtracted for detrending (i.e., centering and equating the means of former and latter trials in ***B***), (4) when trained by even-numbered trials and tested by odd-numbered trials. Note that the conventional sampling of odd-numbered 20 trials (1st, 3rd, 5th, …, 39th) included both former and latter trials as a part and, thus, can be inhomogeneous under baseline drifts. No significant difference was observed among four types of learning in the absence of pure long-term correlations, that is, when both short-term and long-term correlations were absent (left, not significant for all pairs, paired *t* test, 11 sessions with 77 neurons). The significant decrease at the green bar in the presence of pure long-term correlations demonstrates that the long-term correlations do harm for generalization (right, *p < 0.05, paired *t* test, 23 sessions with 189 neurons). The recovery of the classification success by the detrending or the conventional inhomogeneous sampling (trained by even-numbered and tested by odd-numbered trials) suggests that the brain can decode stimulus information under changing environments by using a sophisticated decoder (***p < 0.001, paired *t* test).

To elucidate the impact of short-term correlations, we compared the classification success rates in the absence and presence of pure short-term correlations in Figure 6*A*. For that purpose, we first selected the neurons who have no long-term correlation. That is, we selected the neurons whose baselines did not drift significantly by using the serial correlation statistical test for randomness of fluctuations (Materials and Methods). For those selected neurons, that cannot have long-term correlations, we compared the classification success rates before and after trial shuffling, which was supposed to remove short-term correlations. We computed the classification success rate session by session, as we wanted to include only simultaneously recorded pairs. We found that the impact of pure short-term correlations was small but significantly positive in Figure 6*A*.

Next, as the origin of long-term correlations, we visualized the baseline drifts in Figure 6*B*. For the neurons with significant baseline drifts (to be precise, the same neuron pool as used in Fig. 6*C*, right), we performed the principal component analysis for the average responses to 16 visual stimuli (i.e., tuning curves). The activities of the 189 neurons with baseline drifts were concatenated and transformed (“rotated”) to the same numbers of 189 principal components, from which we chose the first two as the (most informative) axes for visualization. Figure 6*B* plots the average responses (tuning curves) for 1st–20th trials (turquoise blue) and 21st–40th trials (green) separately and demonstrates the baseline drifts over trials shifted the entire activities of neurons. However, it is still unclear, from the simple visualization, whether this drift is, taking form of long-term correlations, significant in decoding.

To elucidate the impact of long-term correlations on decoding, we compared the classification success rates in the absence and presence of pure long-term correlations in Figure 6*C*. Specifically, we compared the cross-validated classification success rates for four types of learning: (1) when trained by former trials and tested by former trials, (2) when trained by former trials and tested by latter trials, (3) when trained by former trials and tested by latter trials after the respective global means were subtracted for detrending (i.e., centering and equating the means of former and latter trials in Fig. 6*B*), (4) when trained by even-numbered trials and tested by odd-numbered trials. Note that the conventional sampling of odd-numbered 20 trials (1st, 3rd, 5th, *…*, 39th) included both former and latter trials as a part and, thus, can be inhomogeneous under baseline drifts. For that purpose, we first selected the neurons who have no short-term correlation. That is, we selected the neurons whose short-term correlation is not significant by using the statistical test accompanying our estimator (Materials and Methods). Note that although the test applies to a pair, we eventually selected the neurons who have no short-term correlation in any pair in the session. For those selected neurons, that cannot have short-term correlations, we compared the classification success rates for the neurons with and without long-term correlations (i.e., statistically significant baseline drifts) as in Figure 6*C*. As a control, no significant difference was observed among four types of learning in the absence of pure long-term correlations, that is, when both short-term and long-term correlations were absent (left, not significant for all pairs, paired *t* test, 11 sessions with 77 neurons). On the other hand, the significant decrease at the green bar in the presence of pure long-term correlations demonstrates that the long-term correlations do harm for generalization (right, *p < 0.05, paired *t* test, 23 sessions with 189 neurons). The recovery of the classification success by the detrending or the conventional inhomogeneous sampling (trained by even-numbered and tested by odd-numbered trials) suggests that the decrease in decoding accuracy is due to the baseline drift (***p < 0.001, paired *t* test). Note that the last two types of leaning may mimic brains’ possible decoding strategies under changing environments, suggesting that the brain could overcome nonstationarity by detrending.

Here, we solely compared the classification success rates obtained for the same neuron pool with different types of learning. This is because we believe that it is dangerous to compare different pools even if the numbers of neurons are equated, as the sensitivity to stimuli varies by neurons, leading to considerable sampling biases. For example, if we compare the two green bars in Figure 6*C*, the classification success rate per neuron trained by former and tested by latter is higher in the presence of long-term correlations (data not shown), suggesting that the overall high classification success in the presence of the long-term correlations can be explained by the sampling biases, i.e., simply because the neuron pool with long-term correlations have more smart neurons.

Taken together, the proposed method enables us to elucidate the impacts of short-term and long-term noise correlations in a dissociated manner. The well-designed decoding analysis with dissociated correlated activities may help to gain insight into the brains’ decoding strategies under changing environments.

## Discussion

In this article, we proposed an information-geometric method to unbiasedly estimate pure short-term noise correlations irrespective of arbitrarily drifting baselines. The simulation demonstrated the robustness of the proposed estimator against the slow, long-term drift. The accompanying statistical test as well as the existing nonstationarity test enabled us to dissociate short-term and long-term correlations. When we exclude the spurious noise correlations of purely long-term nature, only a small fraction of V1 neuron pairs showed significant short-term correlations, possibly reconciling the previous inconsistent observations on existence of significant noise correlations. Finally, with the additional help of the machine learning that classifies stimuli from neural activities, we assessed the impacts on decoding of the presence of short-term or long-term correlations, separately. The presence of pure short-term correlations slightly improved the decoding accuracy, while the pure long-term correlations deteriorated the generalization ability. However, the decrease in decoding accuracy by the long-term correlations was recoverable by using the decoder with offset, suggesting that the brain could overcome nonstationarity by detrending. Thus, our method enables us to elucidate the functions of short-term and long-term correlations in a dissociated manner and the well-designed decoding analysis with dissociated correlated activities may help to gain insight into the brain’s decoding strategies under changing environments.

Our observation that only a small fraction of neuron pairs has short-term noise correlations after detrending may, at first glance, inconsistent with previous works, which reported significant noise correlations. However, the previous works which detrended the time series before calculating noise correlations reported small short-term noise correlations (Bair et al., 2001; Ecker et al., 2010; Renart et al., 2010). In this sense, our result is consistent with the previous results. The previous modeling studies implied that even if short-term noise correlations are small, it can have a big impact in a large network (Zohary et al., 1994; Sompolinsky et al., 2001; Miura, 2012). As far as our V1 dataset, the impact of short-term noise correlations was small but significantly positive.

The classification analysis in this article demonstrated that the presence of baseline drifts decreased the generalizability of the classifier. However, the further analysis showed that the classification success rate can be recovered by detrending data or including more inhomogeneous training data. Note that the generalizability should depend on the training data set: the more different conditions are learned, the higher the classification success rate becomes. In other words, if the future (test) conditions are completely different from the past (learned) conditions, the baseline drifts do harm. Thus, we essentially showed two possible decoding strategies that can overcome nonstationarity. It is interesting to know how the brain decodes visual stimuli from small responses under big spontaneous fluctuations? Can the downstream neurons separate stimuli in the high dimensional space or, alternatively, cancel out the baseline drifts suitably? These questions remained and leave future work, possibly, with well-designed decoding analyses.

In this article, we solely treated spike count correlations as a measure of synchrony. We did not use spike-timing cross-correlograms with milli-second bins (Toyama et al., 1981a,b; Ito et al., 2010) as we took advantage of our proposed method, which is limited to spike count correlations. The limitation comes from the assumption of no temporal auto-correlation in the time series. The assumption is necessary to dissociate short-term and long-term cross-correlations successfully. If we consider millisecond bins, temporal auto-correlations exist, which violates the assumption. Here, we rather focused on spike count correlations to use our proposed method in depth to the extent to elucidate the componentwise functions of short-term and long-term correlations. Thus, we did not say anything on temporal coding in this article, although previous papers suggested the relationship that the spike count correlations increase with coupling strengths (Cossell et al., 2015; Bharmauria et al., 2016).

There are considerable merits for our proposed estimator of short-term correlations. It guarantees the smallest estimation error among all the estimators which “works” for arbitrary baseline drifts. It utilizes differential geometry essentially and otherwise it is generally impossible to cope with infinitely many cases programmatically even with the fastest computers. As a practical advantage, it enables us to perform a statistical test from a single trial or a snapshot of time series with baseline drifts, which is usually unreproducible. The estimating equation given in an analytically closed form as well as the accompanying statistical test, are quite simple and implementable within a few lines of programming codes, easier than shuffling-based methods which have longer lines and computational time. The underlying statistical model allows us not only to test statistical significance but also to interpret the correlation coefficients quantitatively, which is unrealizable for other *ad hoc* or shuffling-based methods.

Meanwhile, to fully exploit the temporal order and continuity of trials without assuming specific statistical models for trends nonparametrically, we had to consider a simplified additive Gaussian noise model. However, some previous works used more realistic models such as mixed Poisson distributions and, for example, estimated the contributions of additive as well as multiplicative noises to explore the underlying biological processes (Goris et al., 2014; Arandia-Romero et al., 2016). Thus, it is desired to pursue temporal structures with more realistic statistical models in the future work.

It is important to check whether the spike count data to be analyzed satisfy the model assumptions of the proposed method. For example, the normality assumption is satisfied by high firing neurons in general due to the central limit theorem. However, strictly speaking, when we checked whether the spike count data used in the article obey the normal distribution by using the Shapiro–Wilk test, only 70.0% of the neuron-stimulus pairs that are stationary (i.e., without drifts) and modest-firing (i.e., more than 5 Hz) satisfied the normality assumption. One possible solution might be to apply the proposed method only to the high firing neurons as low firing neurons tend to violate the normality assumption. If the data do not satisfy the assumption of the normal noises, the proposed noise correlation is no more an optimal parameter estimate of the statistical model. It is also important to check whether the assumption of the constant covariances is satisfied, at least for some time range. For example, strongly nonstationary neurons, whose firing rates grow twofold over an hour, might violate the assumption. We leave the detailed examination of the model assumptions with statistical model selection procedures for the future works. However, if the violation is weak, the proposed measure could still be used as a rough measure. For example, even if the data were actually non-Gaussian spike counts with multiplicative drifts (Goris et al., 2014), the sign of the proposed measure, excitatory or inhibitory, could still be meaningful.

Another assumption for the proposed estimator was that the baseline activities for the consecutive two trials are (almost) the same. This assumption in our analysis was the clue to separate short timescales and long timescales. Strictly speaking, however, as we computed noise correlations separately for different stimuli, the intervals between the trials for the same stimulus are variable. Note that stimuli were presented in a pseudo-random order. In fact, for the worst case, the effective trial interval can be as large as 90 s (3 s × 15 stimulus × 2). Although it is generally hard to characterize the effects of drifts on these medium timescales, no difference was observed between randomized and repeated orders of stimulus presentations (Kohn and Smith, 2005). Thus, we assumed that the drifts on these medium timescales were ignorable. Practically, if the assumption of the constant baseline is doubtful for a trial pair due to the long interval between them, one could remove the pair from the calculation of the proposed estimator. That is, one could exclude unreliable trial pairs from the summation in Equation 2. This type of exception handling could also work for avoiding the change point where the baselines jump suddenly. Developing a more flexible algorithm for the proposed method can be a future work.

10.1523/ENEURO.0266-18.2018.ed1 Supplementary 1 Supplementary R-code. Download Supplementary 1, TXT file

## Synthesis

Reviewing Editor: Tatyana Sharpee, The Salk Institute for Biological Studies Decisions are customarily a result of the Reviewing Editor and the peer reviewers coming together and discussing their recommendations until a consensus is reached. When revisions are invited, a fact-based synthesis statement explaining their decision and outlining what is needed to prepare a revision will be listed below. The following reviewer(s) agreed to reveal their identity: Michael Okun.

The study analyzes how to compute noise correlations between spike counts in trials. This is an important question that received significant attention in the past. It would be helpful to clarify the novel contribution of the method compared to previously proposed approaches, including those based on information-geometry as well as those suggested by both reviewers (e.g. for modulated Poisson processes). Please clarify how specifically information geometry is used to estimate the signal probability distribution. Another point of concern that it is not sufficient to state that the estimator is unbiased because it is normalized by M and not by M-1. There should be a discussion and derivation for the conditions under which the estimator will have zero bias and saturate the Cramer-Rao bound. Finally, please provide the code for confidence interval computations (section 2.5).

Reviewer 1:

In this manuscript the authors apply a previously developed method for the estimation of short time correlations based on detrending to both synthetic and experimentally obtained data sets. They compare the method to other existing approaches, and find that the proposed method offers improved performance. Applications to neural recordings show that, once trends are removed, only a fraction of neuron pairs are significantly correlated, as noted previously by Ecker, et al and others.

Overall, the manuscript may be acceptable. However, the authors need to address a number of issues.

It is unclear why the authors refer to information geometry. It seems that the method uses a local estimate of the rate to avoid the problems that drifting rates introduce into estimates. I did not understand the proof that this estimator is unbiased, or has minimal variance (see below). But the proofs seem elementary, if correct, and did not really rely on information geometry.

Indeed, the approach seems very similar to what one would obtain by just using detrending of the recorded signals using conventional, classical methods - not even Kalman filters. It would be useful to compare the approach with these approaches.

What I find confusing is that Fig. 1 presents correlograms, but the methods section discusses estimates of correlations at a point in time. There are multiple inconsistencies of this type which make the manuscript difficult to understand.

From what I understand the activity of the different cells is the average activity over seconds. But a central assumption is that activity between these windows does not change. I don't see how these assumptions are consistent.

Further comments:

- The very beginning of the description of the proposed method is unclear. What are x and y? Simply stating that this is the activity of a neuron is not sufficient. Is it the rate over some window? How big is the window? Is it the presence of a spike in a time bin? Or something else, or possibly any of these? This is party explained later, I assume, in the statement “the spring activity of a neuron is integrated over a couple of seconds”. However, this is imprecise.

- Which statistical model does the following statement refer to?

“That is, the activities of two neurons were hypothesized to obey the following statistical model and the proposed estimator estimates the Gaussian covariance there in.”

- \xi is usually used for noise. \mu is typically used for the mean. Indeed this notation is used in (13) which makes it even more confusing.

- In Eq. 4, k(\xi) should be denoted as a distribution on the left. Is this a bivariate distribution, ie can x and y have potentially different distributions of means? I assume so, but the notation is unclear.

- I assume that X = (x,y). This was not mentioned anywhere.

- Right above eq. 7, I assume we have \Sigma_12 in the definition of g. I do not understand the next line. Is there a dX missing?

- listing R code in the manuscript is pointless.

- The readability of the manuscript could be significantly improved. While there are no glaring grammatical errors, there are numerous small ones. Some sentences are phrased in a way that makes them difficult to understand. For example,

“Although the pure long-term correlations deteriorated the generalizability, it was recovered by the decoder with trend removal, suggesting that the brain could overcome nonstationarity.”

What does “it” refer to in “it was recovered”? I assume it refers to “generalizability”. However, this sentence was hard to understand.

Another example - the first sentence of the Significance Statement takes considerable effort to parse. In my opinion this is where the author should take particular care to be clear, as the abstract and significance statement will be the two parts of the manuscript that will be read most.

Reviewer 2:

The paper deals with the problem of estimating noise correlations (between pairs of neurons) in a manner that disregards the contribution of slow fluctuations in firing rate. The question has a long history.

There are several major issues that need to be addressed:

* Only towards the end of the Discussion it is clearly stated that the work is about noise correlations between spike counts in trials (each lasting 1-2 seconds, like in the V1 data analyzed), and not between spike trains represented on a much finer scale (e.g. 1 ms windows). This should be stated from the beginning, and then Fig. 1a,b where x-axis shows time (not clear in what units) is confusing - I would presume it should be about trials?

* It is claimed in the Discussion that there is no auto-correlation structure in trial spike counts in the V1 data.

This is very confusing, as my understanding was that any such structure is taken (by definition) to be a manifestation of the long-term drifts. Whether such computationally introduced separation between short-term and long-term correlations actually corresponds to any separate underlying biological processes is not at all clear based on what we know about the mechanisms involved.

* The simulated examples use normally distributed spike counts, and it is claimed that normal distribution is a good approximation for spike counts in trials. In fact this is not the case, and a more realistic distribution would be far more appropriate. For instance, one could use a negative binomial distribution, as in (Goris et al., Nature Neuroscience 2014).

* The aforementioned work of Goris et al. (and related follow up works) provide an elegant solution (albeit with complex fitting procedure) to the very problem the manuscript sets out to address: how to separate between short-term noise correlations and long-term baseline drifts (to the extent such separation actually corresponds to underlying biological processes). It would be quite appropriate for the present manuscript to refer to these works. I don't think that the Kalman filter model (to which the suggested estimator is compared here) is what this recent literature proposes.
